# Dormancy-break and germination requirements for seeds of the threatened Austral papaya (*Carica chilensis*)

**DOI:** 10.1038/s41598-023-44386-y

**Published:** 2023-10-13

**Authors:** Andrea P. Loayza, Patricio García-Guzmán, Giovanni Carozzi-Figueroa, Danny E. Carvajal

**Affiliations:** 1 Instituto Multidisciplinario de Investigación y Postgrado, Universidad de la Serena, 1720256 La Serena, Chile; 2 Instituto de Ecología y Biodiversidad (IEB), 7800003 Santiago, Chile; 3 Facultad de Educación y Ciencias Sociales, Universidad Andres Bello, Santiago, Chile; 4 Departamento de Biología, Universidad de la Serena, La Serena, Chile; 5 Centro de Ciencia del Clima y la Resiliencia, CR2, Santiago, Chile

**Keywords:** Seed development, Plant ecology

## Abstract

Seed dormancy is one of the most important adaptive mechanisms in plants, optimizing germination, seedling emergence, and establishment to ensure these processes occur when environmental conditions are favorable for plant survival and growth. Endemic to rocky environments of the southern Atacama Desert, the Austral papaya (*Carica chilensis*) is the papaya species with the southernmost distribution within the Caricaceae, thriving in the most extreme environmental conditions. This threatened plant exhibits low natural regeneration, primarily attributed to low germination, yet no information regarding seed dormancy release is available. In this study, we investigated the dormancy-break and germination requirements of *C. chilensis*. We hypothesized that if *C. chilensis* seeds exhibit physiological dormancy, then seeds with reduced moisture content and those treated with chemicals or growth hormones would exhibit higher germination percentages and faster germination than control seeds akin to other members of Caricacea. Our results confirmed this prediction and revealed that ultra-drying (< 3% moisture content) and treating seeds with sulfuric acid, gibberellic acid, or potassium nitrate are the most effective methods for germinating *C. chilensis*. Consequently, we suggest using these treatments to propagate this threatened papaya species.

## Introduction

Plants rely on seeds for reproduction, genetic continuity, and colonization^1^. However, the successful establishment of seedlings depends on the timing of seed germination; consequently, seeds must possess strategies to perceive their surrounding environment and trigger germination^2^. Seed dormancy is a plant strategy that optimizes germination, seedling emergence, and establishment so that these processes occur when environmental conditions are favorable for plant survival and growth^2–4^. It refers to inhibited germination in mature seeds even under favorable environmental conditions for germination^5^. Seed dormancy can be particularly important for plants in harsh and highly unpredictable environments (e.g., in arid ecosystems), where windows for recruitment are few and sparse; hence, it is more prevalent in these environments than in those with more benign conditions^6^.

Seed dormancy is classified into five general classes^5^: (1) morphological dormancy (MD), where a seed is immature when the fruit falls and requires a period of growth and embryo differentiation for germination to occur; (2) physical dormancy (PY), where the seed coat is impermeable and prevents water from entering the seed, thus requiring mechanical or chemical scarification for germination to occur; (3) physiological dormancy (PD), where a physiological inhibiting mechanism in the embryo results in low growth potential, which prevents the emergence of the radicle through covering layers, hence seeds require a specific set of conditions (i.e., often a combination of temperature, moisture, and light) to initiate germination; (4) morphophysiological dormancy (MPD), in which an underdeveloped embryo has physiological dormancy, and (5) combinational dormancy, where multiple mechanisms, including physiological, morphological and ecological factors, as well as environmental triggers prevent germination. Most families of plants have seeds with PD^3^, and depending on the strength of the physiological inhibitory mechanism, they can exhibit one of three levels of PD: (1) non-deep (dormancy can be broken with chemicals, gibberellic acid (GA_3_), warm or cold stratification, after-ripening in dry storage, and mechanical or chemical scarification), (2) intermediate (dormancy can be broken after a long period of cold stratification, and GA_3_ may or may not break dormancy), or (3) deep (dormancy can be broken after a long period of cold or warm stratification, and GA_3_ does not break dormancy)^2,3,7–9^.

*Carica chilensis* (Planch. ex A.DC.) Solms., commonly known as the Austral Papaya, is an endemic papaya species of Chile distributed along the southern limit of the Atacama Desert^10–12^. This species belongs to the Caricaceae, a predominantly tropical plant family that includes the common papaya (*Carica papaya*). The family comprises 35 species distributed across six genera^13^, most with seed dormancy^3^. Despite the economic importance of the Caricaceae, studies regarding its germination requirements are scarce, primarily focusing on *C. papaya*^14–16^ or a few *Vasconcella* species^7,8,17,18^. Results from these studies suggest that seeds of this plant family exhibit PD^2,3^ and that low seed germination is due to inhibitory substances, mainly phenolic compounds^14,19–21^, present in both the sarcotesta (the gel-like coating around the seeds) and the sclerotesta (the tough central layer of the testa)^8,9,17^. However, further research is needed to ascertain the kinds of dormancy in Caricaceae, aiming to enhance our comprehension of the factors influencing germination in this family.

This study aimed to investigate the seed dormancy-break and germination requirements of the Austral papaya, the southernmost species of the Caricaceae. We hypothesized that if *C. chilensis* seeds exhibit PD, then seeds with reduced moisture content (MC) and those treated with chemicals or growth hormones would exhibit higher germination percentages and faster germination than control seeds akin to other members of Caricacea^7^. Understanding the mechanisms and factors that govern seed germination and dormancy in *C. chilensis* holds great importance for several reasons. Firstly, the presence of papain in its fruit makes this wild papaya a plant of interest for cultivation^22^. Secondly, *C. chilensis* is classified as Vulnerable due to its limited presence in a few fragmented, low-density populations, and to the decline of natural populations resulting from habitat destruction^11,12,22^. Thus, studies examining its propagation have been recommended as a conservation strategy^23^. Lastly, the lack of knowledge regarding germination requirements of most native non-tree species in Chile is considered one of the major bottlenecks for fulfilling the country’s restoration commitements^24^. Therefore, there is an urgent need for studies assessing the dormancy-break requirements of these species.

## Results

**Figure 1:**
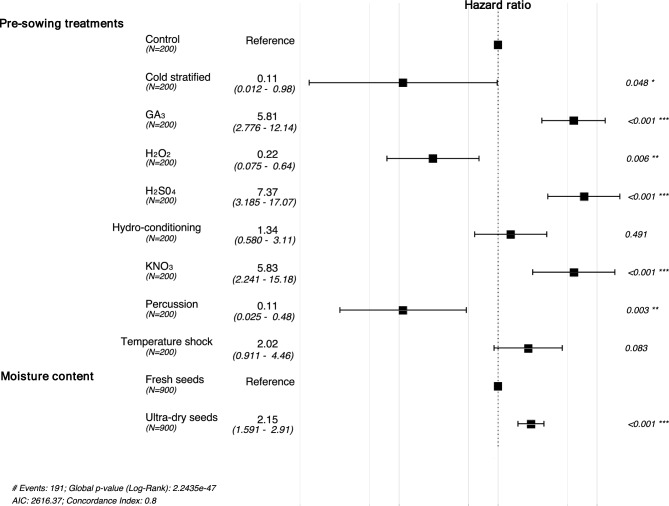
Forest plot of the Cox proportional hazards regressions of germination clustered by Petri dish replicate. The plot shows seed germination probabilities (hazard ratio, HR) and 95% CI from seeds (N) with different moisture content and treated with different pre-sowing treatments. The HR for control and fresh seeds is standardized to 1 and denoted by the dashed vertical line. An HR > 1 indicates an increased germination probability, whereas an HR < 1 indicates a decreased probability.

The moisture content of fresh *C. chilensis* seeds was 33.5% ± 0.98 (mean ± sd). Most essays successfully released seed dormancy; however, the germination percentage remained generally low, varying between 0 and 10% in the pre-sowing treatments for fresh seeds and from 0 to 40% for ultra-dry seeds (Table 1). The germination risk of ultra-dry seeds was 77% higher than that of fresh seeds (Table 2). Pre-sowing treatments significantly influenced the timing of seed germination (χ^2^ = 243.6, gl = 9, *P* < 0.0001; Fig. 1). Specifically, three treatments increased germination probability compared to the baseline germination hazard of control seeds: sulfuric acid (H_2_SO_4_) increased germination by 200%, while both gibberellic acid (GA_3_) and potassium nitrate (KNO_3_) enhanced it by 176% (Table 2).

**Table 1:** Germination percentage of *Carica chilensis* according to seed moisture content (MC) and pre-sowing treatment (mean ± standard error).

MC	Treatment	Germination% Fresh seeds	Germination% Ultra-dry seeds
Fresh	Control	5.0 ± 2.24	4.0 ± 1.63
	Cold stratified	0.00	1.0 ± 1.00
	GA_3_	8.0 ± 3.27	37.0 ± 4.48
	H_2_O_2_	2.0 ± 1.33	0.00
	H_2_SO_4_	17.0 ± 2.60	40.0 ± 7.30
	Hydro-conditioning	9.0 ± 3.40	3.0 ± 1.53
	KNO_3_	13.0 ± 3.0	34.0 ± 8.06
	Percussion	1.0 ± 1.00	0.00
	Temperature shock	10.0 ± 2.58	7.0 ± 3.00

**Table 2:** Results of the Cox proportional hazards model of *Carica chilensis* seed germination with different moisture content (MC) and treated with different pre-sowing treatments.

	Treatment	BETA (SE)	HR (95% CI)	*P*
MC	Fresh seeds (ref)	–	–	–
	Ultra-dry seeds	0.77 (0.15)	2.15 (1.59, 2.91)	< 0.001
	Control (ref)	–	–	–
Pre-sowing treatment	Cold stratified	− 2.21 (1.05)	0.11 (0.01, 0.98)	0.05
	GA_3_	1.76 (0.37)	5.81 (2.78, 12.14)	< 0.001
	H_2_O_2_	− 1.52 (0.78)	0.22 (0.08, 0.64)	0.006
	H_2_SO_4_	2.00 (0.36)	7.37 (3.18, 17.07)	< 0.001
	Hydro-conditioning	0.29 (0.44)	1.34 (0.58, 3.11)	0.49
	KNO_3_	1.76 (0.36)	5.83 (2.24, 15.18)	< 0.001
	Percussion	− 2.21 (1.05)	0.11 (0.03, 0.48)	0.003
	Temperature shock	0.70 (0.41)	2.02 (0.91, 4.46)	0.08

*BETA* β coefficients, *HR* hazard ratio.

**Figure 2:**
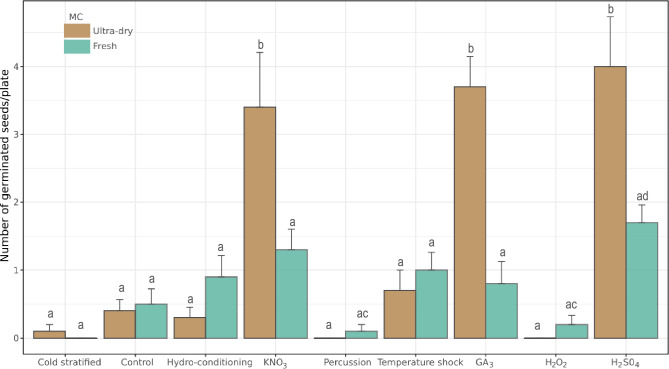
Mean number of germinated seeds per Petri dish based on GLM model for the combined effects of pre-sowing treatments with moisture content (MC). We sowed each Petri dish with 10 *C. chilensis* seeds. Error bars indicate ± SE. Small cap letters indicate differences between the number of germinated seeds among different treatment combinations.

The GLM analysis revealed significant interactive effects of MC and pre-sowing treatments on the mean number of germinated seeds per Petri dish (Table 3). Overall, ultra-dry seeds treated with H_2_SO_4_, GA_3_, or KNO_3_ exhibited the highest germination (Fig. 2).

**Table 3:** Results of the generalized linear model examining the combined effects of moisture content (MC) and pre-sowing treatments on the proportion of germinated seeds of *Carica chilensis.*

Df	Df	Deviance	Resid. Dev	Pr(> Chi)
Pre-sowing treatments	8	21.94	22.56	< 0.001
MC	1	2.50	20.06	< 0.001
MC* Pre-sowing treatments	8	3.62	16.44	< 0.001

## Discussion

Seed dormancy is an adaptive trait that allows plants to delay germination until environmental conditions become favorable for seedling establishment and growth^1,25^; in arid ecosystems, such conditions are generally associated with rainfall events, which are highly variable and largely unpredictable^26,27^. In the plant family Caricaceae, seed dormancy -particularly in the form of PD- is a common characteristic^2,3^. Our findings reveal that over 95% of *C. chilensis* seeds are dormant at maturity and that this dormancy can be partially alleviated by reducing seed MC and treating the seeds with H_2_SO_4_, GA_3_, or KNO_3_. These results suggest that, as predicted, seeds of the Austral papaya have non-deep PD.

Germination was 77% higher in ultra-dry than in fresh seeds. Similar to our results, ultra-drying promoted germination in *Vasconcellea quercifolia*^7^. Reducing seed MC can promote germination via several mechanisms. For example, it can alter seed water potential^28^. In this regard, seeds with lower MC have more negative matric water potentials than seeds with higher MC; as a result, they absorb water more quickly^29^. In contrast, solution uptake by fresh or fully imbibed seeds is driven by differences in osmotic potential between the seed and the surrounding solution^30^. Decreasing MC can also alter seeds’ hormonal balance, reducing abscisic acid (ABA) levels and increasing gibberellins (GA)^31–33^. These two hormones regulate seed germination in opposite manners: ABA promotes seed dormancy and inhibition of germination, whereas GA promotes germination^34^.

A reduction in seed MC is likely to occur naturally in *C. chilensis* because the species produces mature fruits between August and October, prior to the onset of summer. Since both temperature and the gradual loss of MC are integrated over time to alter the depth of the dormancy^2^, the hot and dry conditions of the Mediterranean summer in central Chile likely contribute to the gradual release of dormancy throughout the season. Consequently, seeds can germinate in the field at the onset of the winter rain pulses^35^ and take advantage of the moist soils and warmer temperatures during the spring to promote seedling establishment. Nonetheless, given that germination patterns and seed dormancy can vary geographically^36^ and that we collected seeds from a population located towards the southern end of *C. chilensis*’ distribution, which has drier and warmer summers, it will be informative to confirm if seeds from other localities show similar behavior.

Dormancy was released by treating *C. chilensis* seeds with H_2_SO_4_, GA_3_, or KNO_3_. The seed coat of Caricaceae is multiplicative, consisting of a fleshy outer layer known as the sarcotesta, enclosing a hard, lignified portion of the testa^37^. Hence, scarification with H_2_SO_4_ can enhance water absorption and uptake, essential for initiating germination, by breaking down the seed coat. Gibberellins are a family of plant hormones that control many aspects of plant growth, including germination^2^. GA_3_ stimulates germination through several mechanisms, including producing hydrolytic enzymes that weaken the seed coat^38^, mobilization of nutrient reserves, and stimulation of plant embryo expansion and hypocotyl elongation^33,39^. Numerous studies have documented enhanced germination in *C. papaya* with the application of GA_3_^40^. Similarly, GA_3_ promotes germination in a few species of *Vasconcellea*^7,8^. However, apart from these findings, there is currently a lack of information regarding the effects of GA_3_ in other Caricaceae species. Finally, KNO_3_ can break seed dormancy and promote germination in at least two ways. First, it has hygroscopic properties; when seeds are imbibed in a KNO_3_ solution, they absorb moisture from the surrounding environment, weakening the seed coat. Second, it inhibits ABA synthesis while stimulating the synthesis of GA, which promotes germination by enhancing the growth potential of the embryo and overcoming the mechanical barriers imposed by the testa^2^.

One of the major impediments to the potential use of wild species germplasm for habitat restoration is the need for more knowledge about techniques for breaking seed dormancy and caring for germinating seeds^41^. Thus, improving our understanding of seed biology is crucial to restoring a broader and more representative range of species^42^. Chile is one of 115 countries that has subscribed to restoration commitments^43^. However, dormancy-break and germination requirements of seeds of most non-tree species in Chile remain poorly known. This lack of knowledge hinders the propagation of threatened plants and is recognized as a bottleneck for fulfilling Chile’s restoration commitments^33^. This study contributes to filling this gap with the aim of promoting more extensive use of species diversity in ecological restoration and helping to conserve endangered plant species.

## Methods

### Study species

*Carica chilensis* is an endemic shrub of northern Chile distributed along the southern limit of the Atacama Desert, between the Atacama (28° 39′ S; 71° 42′ W) and Valparaiso (33° 09′ S; 71° 42′ W) Regions. It is classified as threatened, and due to its slow growth and low germination percentages, *C. chilensis* experiences minimal natural regeneration. Like most Caricaeae^13^, this species is polygamous with male, female, and hermaphrodite plants. It produces mature fruits from July to October, with peak fruiting in September. Each fruit contains an average of six seeds enveloped by a gelatinous layer. As the southernmost species in the family, *C. chilensis* is subject to extreme environmental conditions, such as drought and saline soils, across its distribution range.

### Fruit collection site and procedure

We collected mature *C. chilensis* fruits from 40 individuals in the Pupío basin in North Central Chile (32° 07 S, 71° 26 W, 190 masl) between July and September 2021. The area has a Mediterranean climate with warm, dry summers and cool, wet winters^44^. The mean annual temperature is ~ 22 °C; July is the coldest month (~ 9–8 °C, mean coldest temperature), and February is the warmest (~ 22.7 °C, mean warmest temperature). Mean annual precipitation is 227 mm, 86% of which falls from May to August (data from Los Vilos Weather Station, Dirección General de Aguas, 1982–2019).

We collected between five and eight fruits per plant and placed them in paper bags in a cooler until they were processed a maximum of two days later in the laboratory at Universidad de La Serena. We manually removed the seeds from the fruits and cleaned them with a toothbrush under running water to remove the sarcotesta. Then, we pooled the seeds together for the experiments.

### Dormancy-break requirements

We determined the dormancy-break requirements of *C. chilensis* seeds by examining the combined effects of MC and pre-sowing treatments. To establish the MC treatment, we first assessed initial seed MC by weighing 100 seeds before and after drying at 130 °C for three h^45^. Seeds for the ultra-dry treatment were then placed in Eppendorf tubes with blue silica gel and dried to < 3% of their initial MC. We assessed germination of fresh and ultra-dry seeds in each of nine pre-sowing treatments: (1) Control (no pre-sowing treatment); (2) seeds soaked in a 400 ppm solution of gibberellic acid (GA_3_) for 24 h at room temperature; (3) seeds soaked in a 1 M solution of potassium nitrate (KNO_3_) for 30 min; (4) seeds soaked in distilled water changed daily for seven days at room temperature (hydro conditioning); (5) seeds soaked first in distilled water for 24 h and then in hot water (35 °C) for four h (Temperature shock); (6) seeds shaken in a glass jar for 10 min (percussion), (7) seeds cold (moist) stratified, kept at 5 °C for 7 days; (8) seeds soaked in a 100% solution of hydrogen peroxide (H_2_O_2_) for 20 min and; (9) seeds soaked in a 10% solution of sulfuric acid (H_2_SO_4_) for 30 min.

We replicated each treatment combination in 10 Petri dishes and sowed each dish with ten seeds in sterilized sand (60 g), which was hydrated to carrying capacity (*ca.* 12 ml of water). Petri dishes were incubated in germination chambers with 16 h of light at 25 °C^7^, and we monitored germination periodically for 94 days. We considered a seed germinated when the radicle emerged (> 2 mm). At the end of the experiment, we calculated the number of germinated seeds, and the seed germination percentage and rate for each Petri dish.

### Statistical analyses

Initially, we evaluated the independent effects of MC and pre-sowing treatments on seed germination using a Cox Proportional hazards model (‘survival’ package^46^) nested within each Petri dish to consider the non-independence of seeds. In these models, the dependent variable is a hazard function, which represents how the instantaneous “risk” of experiencing the event (i.e., germination) changes with time. This risk function was compared to a "baseline hazard function," representing the risk of the event occurring at a specific time in a reference individual or group^47^. To establish a baseline, we utilized the germination rates of fresh and control seeds as the baseline hazard because this is the natural state of the seeds. By comparing the hazard ratios, we could identify the conditions promoting the fastest germination. Subsequently, we investigated how the combined effects of MC and pre-sowing treatments affect the overall number of seeds germinating using a Generalized Linear Model. In this model, the independent variables were MC and pre-sowing treatment, and the dependent variable was the number of germinated seeds per Petri dish (GLM, family: Poisson, link: log). We performed all statistical analyses using the R statistical environment^48^.

### Relevant legislations, permitting and consent

Plant material collection was carried out per relevant institutional, national, and international guidelines and legislation, including the IUCN Policy Statement on Research Involving Species at Risk of Extinction and the Convention on the Trade in Endangered Species of Wild Fauna and Flora. Seeds were obtained from plants on private lands, and we obtained permission from the landowners to access the areas and collect the seeds. Given that *C. chilensis* is Chile’s sole wild Caricacea species in Chile and easily identifiable in the field, there was no need to collect voucher specimens.

### Supplementary Information

Supplementary Information.

## Data Availability

The datasets used in the current study are available in the Excel file included in the supplementary material section.
